# *In vitro* evaluation of biofilm formation by *Streptococcus mutans* and *Candida albicans* in orthodontic aligners

**DOI:** 10.1590/2177-6709.30.2.e2524192.oar

**Published:** 2025-05-30

**Authors:** Carolina Veronez Garbúggio SANCHES, Raquel Sano Suga TERADA, Adilson Luiz RAMOS, Janaina de Cássia Orlandi SARDI, Maria Gabriely Malveiro ESTEVES, Maria Cristina Bronharo TOGNIM, Sheila Alexandra Belini NISHIYAMA

**Affiliations:** 1 Universidade Estadual de Maringá, Dentistry Department (Maringá/PR, Brazil).; 2 Universidade de Guarulhos, Dentistry Department (São Paulo/SP, Brazil).; 3 Universidade Estadual de Maringá, Department of Basic Health Sciences (Maringá/PR, Brazil).

**Keywords:** Removable orthodontic appliances, Biofilms, Streptococcus mutans, Candida albicans, Aparelhos ortodônticos removíveis, Biofilmes, Streptococcus mutans, Candida albicans

## Abstract

**Introduction:**

Aligners have been used by the orthodontic community for approximately 20 years, but little research has been carried out on the accumulation of biofilm on the surface of these aligners, as well as their possible impact on the oral ecosystem.

**Methods:**

Ten hemi-arches of Invisalign® brand orthodontic aligners were used. The hemi-arches were placed inside sterile flasks containing 25 mL of Gibbons and Nygaard broth, with standardized suspensions of the two microorganisms on the 0.5 MacFarland scale and incubated in aerophily (*C. albicans*) and microaerophily (*S. mutans* and mixed biofilm) at 37°C for 72h. The biofilm formed was removed by the multiple rinses method to quantify the microorganisms in the biofilms in CFU/mL. A qualitative analysis with scanning electron microscopy was performed to observe the structure of the formed biofilms.

**Results:**

It was observed the accumulation of a monospecies biofilm (*S. mutans* - 2.55 x 10^6^ and *C. albicans* - 1.62 x 10^7^) and mixed biofilm (*S. mutans* - 2.21 x 10^5^ and *C. albicans* - 1.06 x 10^7^) very robust on the surface of orthodontic aligners.

**Conclusion:**

According to the results obtained, one can conclude that Invisalign® brand orthodontic aligners are susceptible to the accumulation of monospecies and mixed biofilms of *S. mutans* and *C. albicans*. Therefore, it is necessary to consider the possibility of Invisalign® users developing carious lesions associated with the biofilm formed by these two microbial species, and to guide the patient on the correct cleaning of the device during treatment.

## RESUMO

**Introdução:**

Os alinhadores têm sido utilizados pela comunidade ortodôntica há aproximadamente 20 anos, mas poucos estudos têm sido realizados sobre a formação de biofilme na superfície desses alinhadores, bem como o seu possível impacto no ecossistema oral.

**Métodos:**

Foram utilizados dez hemiarcos de alinhadores ortodônticos da marca Invisalign®. Os hemiarcos foram colocados dentro de frascos estéreis contendo 25mL de caldo Gibbons e Nygaard, com suspensões padronizadas em 0,5 na escala de McFarland dos microrganismos *Streptococcus mutans* (incubado em microaerofilia) e *Candida albicans* (incubado em aerofilia), tanto sozinhos para formação de biofilme monoespécie, quanto associados, para a formação do biofilme misto (incubado em microaerofilia). O biofilme formado foi removido pelo método de enxágues múltiplos, a fim de quantificar os microrganismos aderidos nos biofilmes, em CFU/mL. Foi realizada uma análise qualitativa com microscopia eletrônica de varredura para observar a estrutura dos biofilmes formados.

**Resultados:**

Foi observado o acúmulo de um biofilme monoespécie (*S. mutans* = 2,55x 10^6^ e *C. albicans* = 1,62 x 10^7^) e misto (*S. mutans* = 2,21 x 10^5^ e *C. albicans* = 1,06 x 10^7^) muito robusto na superfície dos alinhadores ortodônticos.

**Conclusão:**

De acordo com os resultados obtidos, pode-se concluir que os alinhadores ortodônticos da marca Invisalign® são suscetíveis ao acúmulo de biofilmes monoespécies e misto de *S. mutans* e *C. albicans*. Portanto, é necessário considerar a possibilidade dos usuários do Invisalign® desenvolverem lesões cariosas associadas ao biofilme formado por essas duas espécies microbianas, e orientar o paciente sobre a correta higienização do aparelho durante o tratamento.

## INTRODUCTION

The number of adult patients seeking orthodontic treatment has increased significantly and, with it, the need to develop more aesthetic orthodontic solutions to meet the growing social and cosmetic demands. Thus, in 1997, the Invisalign^®^ system emerged, which aims to align teeth in a more predictable and comfortable way, using precise “invisible” plastic retainers to achieve tooth movement, thus meeting the aesthetic demands of patients. 

Although these aligners have been used by the orthodontic community for almost 20 years, there is still little research conducted on these appliances and how much they can disrupt the oral microbiota and promote biofilm accumulation. It is recognized that the oral cavity is colonized by a very complex microbiota, with several microbial species coexisting in different niches that establish homeostasis with the host.^1^ However, environmental changes can impact the local microbial composition, resulting in dysbiosis and enabling the development of diseases.^2^

Many microorganisms present in the oral cavity have the potential to cause diseases in opportune situations. It is known that most oral diseases are associated with microbial biofilms.^3^ Therefore, maintaining oral health, especially when controlling the most common clinical manifestations associated with biofilms (dental caries, periodontal diseases and oral candidiasis), is essential to understand the formation and composition of dental biofilms in order to find possible strategies that can be adopted for their control.^4^

The use of removable appliances for relatively short periods can result in bacterial biofilm accumulation on the tooth’s surface and on acrylic bases.^5^ It has been suggested that removable appliances favor local adherence and colonization of *Streptococcus mutans* ^6^ and *Candida albicans*,^7^ whose presence has been related to the appearance of caries lesions,^6^ reddish coloration of the mucosa, discomfort, halitosis and taste alteration.^8^ Thus, the growth of biofilms on the surface of these appliances can compromise the oral health of patients and compromise the effectiveness of orthodontic treatment.^9^

In this context, orthodontic aligners are also abiotic surfaces that can facilitate the accumulation of biofilm.^10^ However, as far as we know, there is still no research aimed at investigating and quantifying the nature of the biofilms adhered to this type of appliance. Considering the long duration of this type of treatment, which can last for up to 42 months,^11^ there is a concern to satisfy the aesthetic requirements, with the correct alignment of the teeth, but without sacrificing the patient’s health. Thus, the objective of this study was to evaluate the *in vitro* formation of single and mixed biofilms of *Streptococcus mutans* and *Candida albicans* in orthodontic aligners.

## MATERIAL AND METHODS

### STUDY DESIGN

This is an *in vitro* study in which triplicate experiments were performed to evaluate the possible formation and quantification of single and mixed biofilms composed of *Streptococcus mutans* and *Candida albicans* in orthodontic aligners. A complementary qualitative analysis by Scanning Electron Microscopy (SEM) was performed.

### *IN VITRO* EXPERIMENT FOR BIOFILM QUANTIFICATION

#### Sample preparation

For the *in vitro* experiment, 10 hemi-arches of Invisalign^®^ orthodontic aligners were analyzed (Lot 6671479 from the year 2018). The hemi-arches were made using a 7020 double-faced flex diamond disc (KG Sorensen) in a straight piece and Sof-Lex™ Pop-On (3M) abrasive discs on a micromotor. Then, the aligners were sent for ethylene oxide sterilization.

#### Microorganisms

*Streptococcus mutans* (ATCC 25175) and *Candida albicans* (ATCC 10231) strains were used and cultivated in BHI broth at 37°C for 48 hours in microaerophilia for *S. mutans* and in aerobiosis for *C. albicans*. After cultivation, an aliquot of each culture was submitted to Gram stain, for the analysis of the morphological and staining characteristics of the microorganisms and pure culture.

#### Biofilm formation

In a glass jar containing 25 mL of previously sterilized Gibbons and Nygaard broth,^12^ a hemi-arch of the orthodontic aligner was aseptically introduced for the formation of monospecies and mixed biofilms. Subsequently, standardized inocula of the *Streptococcus mutans* (which were incubated in microaerophilia) and *Candida albicans* (which were incubated in aerobiosis) strains were added on a 0.5 McFarland scale (1.5 x 10^8^cells/mL) at 37°C for 72 h. The mixed biofilm was performed under microaerophilia conditions.

#### Biofilm quantification

After the formation of the monospecies and mixed biofilms, each hemi-arch of the orthodontic aligner was carefully rinsed in a tube containing 10 mL of sterile saline solution (NaCl, 0.9%), to remove the cells that were not adhered.

Then, the biofilm was removed by multiple rinses, where each hemi-arch was inserted into a tube (Tube 1) containing 10 mL of sterile saline solution and vortexed (Scientific Industries Inc., New York, USA) for 60 seconds. The procedure was repeated two more times, with the devices transferred to different tubes (Tubes 2 and 3) in sequence, under the same conditions. After removing the biofilm, each tube was diluted three times (10^-1^, 10^- 2^, 10^-3^) and 20 μL of the obtained dilutions were plated by the modified drop method in triplicate.

For plating and counting the colonies of the monospecies biofilm, Mitis Salivarius Agar (Difco, New Jersey, USA) was used for *S. mutans* and Sabouraud Agar (Difco, New Jersey, USA), for *C. albicans*. For the mixed biofilm analysis, 2 μg/mL of amphotericin B were added to Mitis Salivarius Agar and 2 μg/mL of ampicillin, to Sabouraud Agar, to inhibit the growth of *C. albicans* and *S. mutans*, respectively. The concentrations of antimicrobials used were based on the European Committee on Antimicrobial Susceptibility Testing (EUCAST - version 10, 2020).

**Figure 1:**
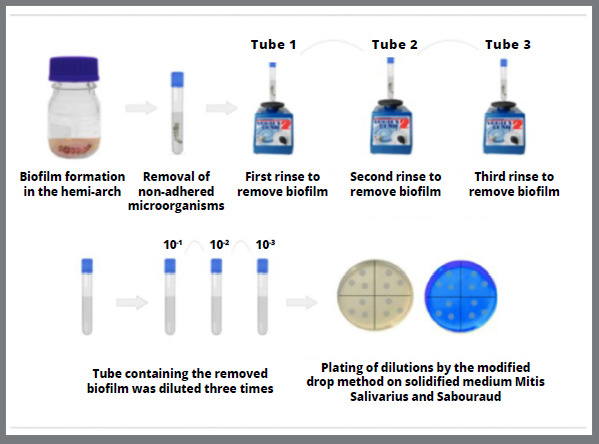
Flowchart of the experimental design of the present study.

Next, the seeded plates were incubated at 37°C for 48 hours in aerobiosis for *C. albicans* and in microaerophilia for *S. mutans* and for the mixed biofilm, for colony count and determination of the CFU/mL of each microorganism (Fig. 1).

### MORPHOLOGICAL AND STRUCTURAL ANALYSIS OF THE BIOFILM IN SCANNING ELECTRON MICROSCOPE

For the qualitative analysis of the biofilm by SEM, the following were performed: samples with a maximum size of 1 x 1 cm were taken of the aligners, with three samples for the mixed biofilm and three samples of the same size for each microorganism in the monospecies biofilms. For the preparation of such samples, 7020 flexible diamond discs (KG Sorensen) were used in a straight workpiece, and Sof-Lex™ Pop-On (3M) abrasive discs in a micromotor. Once prepared, the aligner samples were sterilized using ethylene oxide before the experiment. 

Sample preparation was developed according to Weber et al.^13^ and Bodelón et al.^14^, with some modifications. Biofilms were fixed with 1 ml of glutaraldehyde 2.5% in sodium cacodylate buffer 0.2 M (pH 7.2 - 7.4) for 1h at room temperature. The samples were dehydrated with an increasing series of anhydrous alcohol (PA) concentrations: 50%, 70%, 80%, 90%, 95% and 100%, for 15 min in each concentration, and repeated three times in the pure alcohol step, to ensure the complete removal of water.

Subsequently, the samples were transferred to the critical point system (Bal-TEC CPD 030), mounted on aluminum disks (STUB) and glued with double-sided carbon adhesive tape, in order to be coated with a layer of gold (20 nm) using a metallizer (Bal-TEC SCD 050). The analysis was performed using a Quanta 250 scanning electron microscope (Thermo Fisher Scientific, MA, USA), and the images were generated at four levels of magnification: 1,000x, 2,000x, 5,000x and 10,000x.

### STATISTICAL ANALYSIS

All data obtained were analyzed by ANOVA test and by Tukey’s post-test. Analyzes were performed in GraphPad Prism 5.0, adopting a confidence level of 95% (*p*<0.05) for all analyses. The number of microorganisms was compared in the monospecies biofilm and mixed, between species (*C. albicans* with *S. mutans*) and intraspecies (*C. albicans* in the monospecies biofilm with *C. albicans* in the mixed biofilm and *S. mutans* in the monospecies biofilm with *S. mutans* in the mixed biofilm).

## RESULTS

### BIOFILM QUANTIFICATION

**Figure 2:**
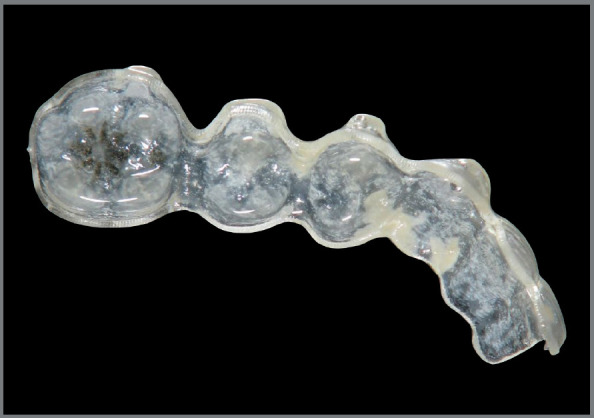
Mixed biofilm formation of *S. mutans* and *C. albicans* in the hemi-arch of the Invisalign^®^ orthodontic aligner.

**Figure 3:**
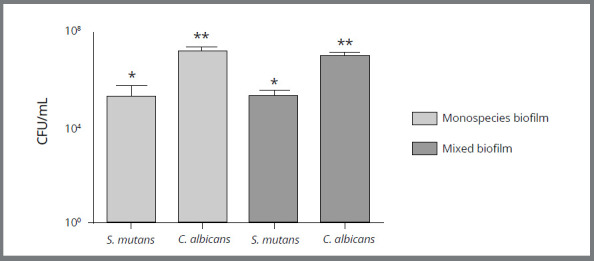
Quantification of colonies in biofilms formed in hemi-arches of Invisalign^®^ aligner (*in vitro* study). CFU/mL values of *S. mutans* and *C. albicans* organized in monospecies and mixed biofilms. ANOVA test and Tukey post-test. *Different graphic signals represent statistically significant differences (*p*<0.05).

By means of the *in vitro* experiment using the Invisalign^®^ hemi-arches, it was observed that the microorganisms *S. mutans* and *C. albicans* formed a thick layer of monospecies and mixed biofilm (Fig. 2) within 72 h of incubation and the CFU/mL was determined from the average colony count in triplicate. The*S. mutans* biofilm presented an average amount of 2.55 x 10^6^CFU/mL in the monospecies biofilm and 2.21 x 10 ^5^CFU/mL when in a mixed biofilm. This was significantly lower ( *p*<0.05) than *C. albicans* , which presented an average of 1.62 x 10^7^ CFU/mL in monospecies biofilm and 1.06 x 10 ^7^ CFU/mL when in mixed biofilm. Such a number represents a quantity ten to one hundred times greater than the bacterium ( Fig. 3). The monospecies or mixed biofilm of the isolated microorganisms did not present a statistically significant difference, only when the number of CFU/mL was compared between them.

### MORPHOLOGICAL AND STRUCTURAL ANALYSIS OF THE BIOFILM

**Figure 4:**
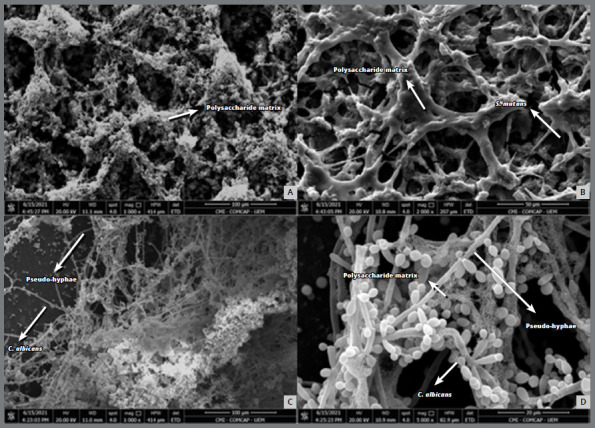
Photomicrographs obtained by scanning electron microscopy of monospecies biofilms formed on the surface of the Invisalign^®^ aligner. **A)** Overview of the *S. mutans* biofilm, which has a large amount of polysaccharide matrix (10,000x). **B)** Close-up view of the *S. mutans* biofilm, showing the polysaccharide matrix tangle and chains of *S. mutans* (2,000x). **C)** *C. albicans* biofilm overview (1,000x). **D)** Close-up view of the *C. albicans* biofilm, showing yeasts, pseudohyphae and polysaccharide matrix (5,000x).

**Figure 5:**
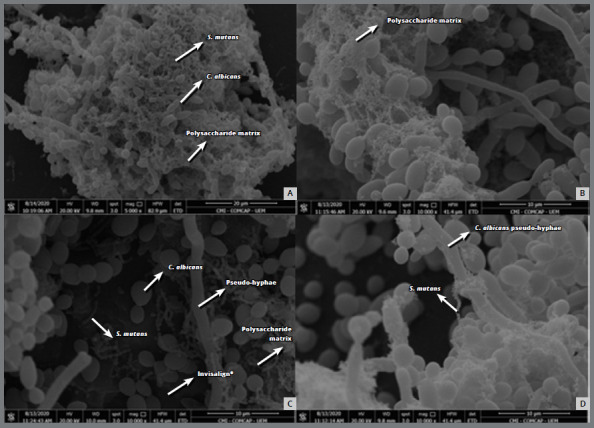
Photomicrographs obtained by scanning electron microscopy of the mixed biofilm formed on the surface of the Invisalign^®^ aligner. **A)** Overview of the mixed biofilm, which has a large biomass of *S. mutans* and *C. albicans* and a polysaccharide matrix (5,000x). **B)** Polysaccharide matrix (10,000x). **C)** Relationship of the microorganisms *S. mutans* and *C. albicans* with the surface of the Invisalign^®^ aligner (10,000x). **D)** Close relationship of *S. mutans* and *C. albicans* microorganisms (10,000x).

A qualitative analysis of the biofilm’s morphology for the Invisalign^®^ hemi-arches was performed by SEM. The SEM for the monospecies biofilm of *S. mutans* showed that this microorganism formed clusters that were incorporated into extracellular polymeric substances (EPS), which were not seen in mixed biofilms, due to the “naked” appearance (without the EPS matrix) of the *S. mutans* cells (Fig. 4). The photomicrograph of the monospecies biofilm of *C. albicans* showed that this microorganism formed a much higher number of pseudohyphae when compared to the mixed biofilm. The mixed biofilm revealed a large biomass of microorganisms, in which the presence of the functional matrix formed by extracellular polymers, the adhesion and relationship of microorganisms, and the formation of pseudohyphae in smaller numbers in the yeasts can be observed, when compared to the monospecies biofilm (Fig. 5). 

## DISCUSSION

In the present study, it was possible to observe the formation of a robust biofilm both in experiments with only one of the microorganisms and in the mixed biofilm of *S. mutans* and *C. albicans* in the Invisalign^®^ orthodontic aligners. These results corroborate the findings of Low et al.,^10^ demonstrating that the surface of the studied orthodontic aligners is susceptible to biofilm formation. We emphasize, however, that the biofilms in this work were developed *in vitro* with the strains of *S. mutans* and *C. albicans*, and the quantification of the attached microorganisms was performed after the formation of the biofilm. 

It is recognized that the presence of new abiotic surfaces in the oral cavity, such as clear aligners, facilitates the accumulation of biofilms. The devices can become reservoirs for microorganisms, some with opportunistic characteristics, which represents a greater risk for oral infections and, consequently, to the health of patients.^9^

Recent molecular studies have modified the conventional view on the pathobiology of dental caries. The predominance of classical cariogenic prokaryotes such as *S. mutans* has been questioned, suggesting the participation of other microorganisms in the carious process, including eukaryotes such as *C. albicans*.^15^ ^,^ ^16^ Although bacterial biofilms are extensively studied, few studies have addressed fungal-bacterial biofilms and the possible microbial interactions that may determine virulence in mixed biofilms in dental caries. To the best of our knowledge, there are no published studies on biofilm formation of *S. mutans* and *C. albicans* in the role of carious lesions in patients using orthodontic aligners.

The participation of *S. mutans* is recognized in dental caries and is considered the main etiologic agent. Its virulence is also related to the ability to generate an acidogenic niche that goes beyond the salivary buffer capacity, triggering changes in the mineral layer of the tooth’s surface, causing progressive demineralization and initiating the carious process.^17^ *S. mutans* is a primary colonizer of dental surfaces, due to its high adhesion capacity associated with the production of a heterogeneous group of proteins, such as glucan-binding proteins (GBPs), that promote its adhesion to tooth surfaces. Furthermore, they produce an extracellular polysaccharide (EPS) matrix, which consists mainly of glucan and fructan from the activity of the exoenzymes glycosyltransferases (Gtfs) and fructosyltransferases (Ftf), respectively, which favor the formation of biofilms.^18^

The ability to synthesize EPS from *S. mutans* can be well observed in monospecies biofilm. Cell clusters incorporated into an EPS matrix can be seen in this work in the photomicrographs obtained by SEM. However, in mixed biofilms with *C. albicans,* it is more common to see “naked” *S. mutans* cells, that is, without the EPS matrix around them. The rapid consumption of sucrose, observed after 10 h in a biofilm of *C. albicans* and *S. mutans* in the study by Sztajer et al.^18^ may explain the reduction in the synthesis of EPS matrix in the mixed biofilm, due to the substrate depletion and, consequently, inactivity of the Gtf and Ftf enzymes. However, the interactions between these species are more complex. A study carried out by Bachtiar and Bachtiar^15^ showed that *C. albicans* contributed to the increase in the expression and concentration of Gtf by *S. mutans* in mixed biofilms. The Gtf produced by *streptococci* favors the production of large amounts of EPS *in situ* using sucrose as a substrate, promoting adhesive interactions and biofilm development between these microorganisms^19^. There is still a Gtf-mediated cross-feeding mechanism that benefits *C. albicans* by providing readily metabolizable monosaccharides, promoting fungal growth and acid production. The change in environmental pH, on the other hand, may favor the survival of *S. mutans,* because it is aciduric.^20^

It is also suggested that *C. albicans* develops a symbiotic coexistence with *S. mutans* in an environment rich in sucrose, increasing the production of lactic acid and reducing the release of mutacins, a protein that interferes with the invasion and proliferation of other bacteria in the biofilm. This could influence the bacterial composition of biofilms formed when *C. albicans* is present and modulate its pathogenic potential.^21^

In turn, the cariogenic potential of *C. albicans* is related to the fact that it is dentinophilic, aciduric and acidogenic, capable of assimilating and fermenting dietary sugars, producing collagenolytic proteases and dissolve hydroxyapatite. A study by Nikawa et al.^22^ showed that even in lower *S. mutans* numbers, the yeast was able to dissolve hydroxyapatite at a rate 20 times higher, suggesting a greater cariogenic potential, particularly in dentin and root cementum. Like *S. mutans*, this yeast has a high ability to adhere to abiotic surfaces, such as denture bases or oral appliances, especially on irregular surfaces with microporosities, cracks and grooves, where it follows the cavity crevices, a property known as thigmotropism.^23^ Furthermore, the hydrophobicity of its cell surface plays an important role in adhesion to inert surfaces and the formation of robust biofilms.

Biofilm formation and virulence of *C. albicans* are also related to the transition of the morphotype of this microorganism from yeast to pseudohyphae. Yeast cells predominantly colonize surfaces, while the pseudohyphal form of *C. albicans* is invasive,^19^ providing structural integrity to the biofilm, which may hinder the host defense mediated by phagocytic cells and stimulate the production of pro-inflammatory cytokine than the yeast-like form.^24^ However, in simple biofilm there is a greater number of *C. albicans* pseudohyphae, as we can see in SEM in this study. According to Jarosz et al.,^25^ this occurs because during the initial stages of growth, *S. mutans* is able to secrete a competence-stimulating peptide, a quorum sensing molecule, which inhibits the transition of the pseudohyphal morphotype of *C. albicans.*

*S. mutans* is considered an efficient microorganism in adapting to environmental stresses, persisting in the host and competing with other oral microorganisms, particularly when conditions are favorable for the initiation and progression of dental caries, which confers a significant ecological advantage.^26^ However, we found an amount of *C. albicans* 100 times greater than that of *S. mutans* in the mixed biofilm, which was also 10 times greater in the simple biofilm, when compared to the amount of this bacterium in the mixed biofilm. As proposed by Jakubovics,^27^ the first dental biofilm colonizers can promote the establishment of other species, which become more dominant as the biofilm develops. Brusca et al.^28^ evaluated the mechanisms of microbial interaction between *S. mutans* and *C. albicans* through the growth kinetics of these microorganisms, when cultivated individually or associated for 36 hours. The authors showed that the initial growth rates of both species were positively influenced by their mutual interaction, however, *C. albicans* prevented bacterial growth at longer incubation times of 36 hours. Thus, during co-colonization, a synergistic beneficial process operates between these microorganisms in short times, followed by competition in longer times, as tested in this study, where *S. mutans* was finally overcome by *C. albicans* after cultivation for 72 hours.

When we associate the characteristics of microorganisms to the fact that patients wear aligners for approximately 22 hour a day, that salivary flow becomes limited and that self-cleaning activities of orofacial soft tissues are interrupted, the development of a biofilm under the aligners is of concern.^29^ This condition can lead to the development of white spots on the enamel and to tooth decay.^30^ Therefore, with all these findings, we need to consider the possibility of indicating to those patients using Invisalign^®^ and who develop carious lesions, how to perform the correct hygiene during treatment.

## CONCLUSION

According to the methodology used and the results obtained in this study, we can conclude that Invisalign^®^ orthodontic aligners are susceptible to the accumulation of *S. mutans* and *C. albicans* biofilm. Therefore, it is necessary to consider that patients using Invisalign^®^ can potentially develop carious lesions associated with simple or mixed biofilm formed by these two microbial species. Thus, it is important to guide the patient regarding the correct hygiene of the device during treatment.
